# 

**Published:** 1909-02

**Authors:** 


### BOOKS RECEIVED.

Constipation and Intestinal Obstruction (Obstipation).. By Samuel Goodwin Gant, M.D., LL.D., Professor of Diseases of the Rectum and Anus, in the New York Post-graduate Medical School and Hospital; attending surgeon for Rectal Diseases at the New York Post-graduate and Saint Mary's Hospital, and the German Polyclinic Dispensary, with 250 original illustrations. Philadelphia and London: W. B. Saunders Company. 1909. (Price, $6.00 net).

American Practice of Surgery. A complete System of the Science and Art of Surgery by representative surgeons of the United States and Canada. Edited by Joseph D. Bryant, M.D, and Albert H. Buck, M.D., New York. Complete in eight volumes. Vol. IV. Imperial octavo, pp. 1010. Profusely illustrated. New York: William Wood & Company. 1909. (Price, cloth, $7.00.)

Saunders's Pocket Medical Formulary. With an appendix containing many useful tables, and suggestions for treatment in emergency conditions. By William M. Powell, M.D., member of the Philadelphia pathological society, etc. Ninth editon, thoroughly revised, and enlarged. Philadelphia and London: W. B. Saunders Company. 1909. (Price, $1.75 net).

Report of the United States Commissioner of Education for the year ended June 30, 1907. Volume II. Washington: Government Printing Office. 1908.

The Urine and Clinical Chemistry of the Gastric Contents, the Common Poisons and Milk, by J. W. Holland, M.D., Professor of Medical Chemistry and Toxicology in Jefferson Medical College, Philadelphia. Forty illustrations. Eighth edition, revised and enlarged. Philadelphia: P. Blakiston's Son & Co. 1908. (Price, $1.00).

A Textbook of Genitourinary Diseases, including Functional Sexual Disorders in Man by Dr. Leopold Casper, professor in the University of Berlin. Translated and edited by Charles W. Bonney, B.L., M.D., Assistant Demonstrator of Anatomy in Jefferson Medical College, Philadelphia, etc. Second edition revised and enlarged with 23 illustrations and 24 full page plates, of which 8 are in colors. Philadelphia: P. Blakiston's Son & Co. 1909. (Price, $5.00).

